# *Schima superba* outperforms other tree species by changing foliar chemical composition and shortening construction payback time when facilitated by shrubs

**DOI:** 10.1038/srep19855

**Published:** 2016-01-27

**Authors:** Nan Liu, Qinfeng Guo, Hai Ren, Zhongyu Sun

**Affiliations:** 1 Key Laboratory of Vegetation Restoration and Management of Degraded Ecosystems, South China Botanical Garden, Chinese Academy of Sciences, Guangzhou 510650, China; 2 USDA FS, Eastern Forest Environmental Threat Assessment Center, Research Triangle Park, NC 27709, USA; 3 Guangdong Open Laboratory of Geospatial Information Technology and Application, Guangzhou Institute of Geography, Guangzhou, 510070, China

## Abstract

A 3.5-year field experiment was conducted in a subtropical degraded shrubland to assess how a nurse plant, the native shrub *Rhodomyrtus tomentosa*, affects the growth of the target trees *Pinus elliottii*, *Schima superba*, *Castanopsis fissa*, and *Michelia macclurei*, and to probe the intrinsic mechanisms from leaf chemical composition, construction cost (CC), and payback time aspects. We compared tree seedlings grown nearby shrub canopy (canopy subplots, CS) and in open space (open subplots, OS). *S. superba* in CS showed greater growth, while *P. elliottii* and *M. macclurei* were lower when compared to the plants grown in the OS. The reduced levels of high-cost compounds (proteins) and increased levels of low-cost compounds (organic acids) caused reduced CC values for *P. elliottii* growing in CS. While, the levels of both low-cost minerals and high-cost proteins increased in CS such that CC values of *S. superba* were similar in OS and CS. Based on maximum photosynthetic rates, *P. elliottii* required a longer payback time to construct required carbon in canopy than in OS, but the opposite was true for *S. superba*. The information from this study can be used to evaluate the potential of different tree species in the reforestation of subtropical degraded shrublands.

Forest disruption and resultant erosion of biodiversity is one of the most damaging human-made changes to the planet^1^,^2^. From 2000 to 2010, for example, nearly 130 million hectares of forest (representing 3.2% of the total forest area in 2000) were lost worldwide^3^. In South China alone, an estimated 3.74 × 10^6^ ha that once possessed by native forests is now degraded and without desirable forest tree species, and natural succession from degraded shrubland to regional climax forest is slow and may require hundreds of years^4^. To accelerate this process, researchers have attempted many methods including the use of nurse plants^5^,^6^,^7^,^8^,^9^,^10^.

By definition, nurse plants facilitate the establishment and growth of other plant species (target species) under their canopies by providing a benign microhabitat condition^11^. Shrubs are found to be ideal nurse plants because they have strong facilitative effects on the survival and initial growth of certain target species^6^,^12^. The shade condition under shrub nurse plant may protect target species from high temperature and radiation, as well as improve soil moisture and nutrition (i.e., fertility islands) and target plant water relations^7^,^8^,^9^. Nurse plant coverage may well facilitate tree seedlings in their early growth because most tree species (late successional species) are shade tolerant in juvenile stage but grow in higher light conditions as they mature^6^,^13^,^14^. In contrast, the fast growing pioneer tree species show complex differentiation in response to light environments, and the canopy shading gradients may affect the growth of pioneers in different patterns^15^,^16^. Therefore, even the ameliorated microenvironmental factors under canopies are important for plant establishment, tree seedlings may not always respond positively to the improved environment in terms of establishment and growth. Our previous studies conducted in subtropical shrubland also showed that all the tree seedlings exhibited improved growth under shrub nurse plant in the first growing season^8^, but their performances varied largely in the following growing seasons due to changes in environmental factors^17^. Although soil properties, and plant photosynthetic parameters have been measured and used to explain target plant growing performance, the intrinsic mechanisms (e.g. alterations in plant chemical compositions and redistribution of photosynthates) underlying target plant responses are still incompletely understood. As the rhizosphere nutrient accumulation and cycling processes accelerate beneath nurse plant canopy, the carbon status and chemical compositions of the target species can be modified^7^,^8^,^11^,^12^.

Allocation of assimilated carbon and mineral nutrients has proven to be a key process that regulates biosynthesis and conversion of sugar into different chemical compounds in each organ of the plant^18^. To understand such carbon investments, researchers assess the biosynthetic pathways leading to the construction of a plant organ and calculate the construction cost (CC), which is defined as the amount of glucose required to produce 1 g of biomass. In previous studies, CC values were obtained from relatively quick measurements, based on strong correlations between the CC values of organic compounds and either carbon concentrations or heat of combustion values^19^,^20^. Such analyses, however, do not provide insights into the causes underlying differences in CC values. Better understanding of the variation in CC values of woody species will require the determination of how the chemical composition of leaves is related to the observed variation in CC values^18^.

Based on their chemical composition and CC values, plant compounds can be classified into eight principal groups^18^,^21^,^22^. Researchers have estimated that plants require 2–3 g of glucose to produce 1 g of the following four groups: lignin, protein, soluble phenolics, and lipids^23^. The production of 1 g of the following three groups requires approximately 1 g of glucose: total structural carbohydrates (TSC), total non-structural carbohydrates (TNC), and organic acids. The eighth group consists of minerals, which are considered to require no glucose except that needed to support the root respiration involved in mineral absorption^21^. The payback time required for photosynthesis to recover CC values, also an important factor for understanding carbon investments^24^, is defined as the time that a leaf requires to amortize its cost, i.e. the time that a leaf must photosynthesize to recover the cost invested in its construction^25^.

Although previous studies have shown that biological and environmental factors (e.g. light environments, species-specific traits, and pollutants) can alter the cellular concentrations in leaves of membrane lipids, pigments, proteins, phenolics, mineral nutrients, and lignin, as well as CC values^22^,^25^,^26^, the plant nurse effects on CC values and on leaf chemical compounds have not been studied. Despite our improved understandings of target plant physiological performance in response to shrub nurse effects^8^,^9^,^12^, we still know little about the carbon metabolism of target plant tissues.

Here, we describe a field experiment that lasted 3.5 years and that involved different target tree species planted under shrub canopies and in open areas in a subtropical degraded shrubland. Our general goal is to increase our understandings on the mechanisms underlying shrub nurse effects on target species. Specifically, we examine how growth, physiological traits, leaf chemical compositions, and CC values of four target plant species responded to a selected nursing shrub plant after 3.5 years. We aim to test our hypotheses in this study: (1) the growth of the fast growing species are suppressed under the canopy of shrub while the more shade tolerant species are facilitated; (2) changes in foliar chemical composition, CC values and payback time may help explore the intrinsic mechanisms in such differences. We also consider the implications of species-specific differences in growth, CC values, and payback time for the reforestation of subtropical degraded shrubland in South China.

## Results

### Plant growth, photosynthetic and structural parameters

**Figure 1:**
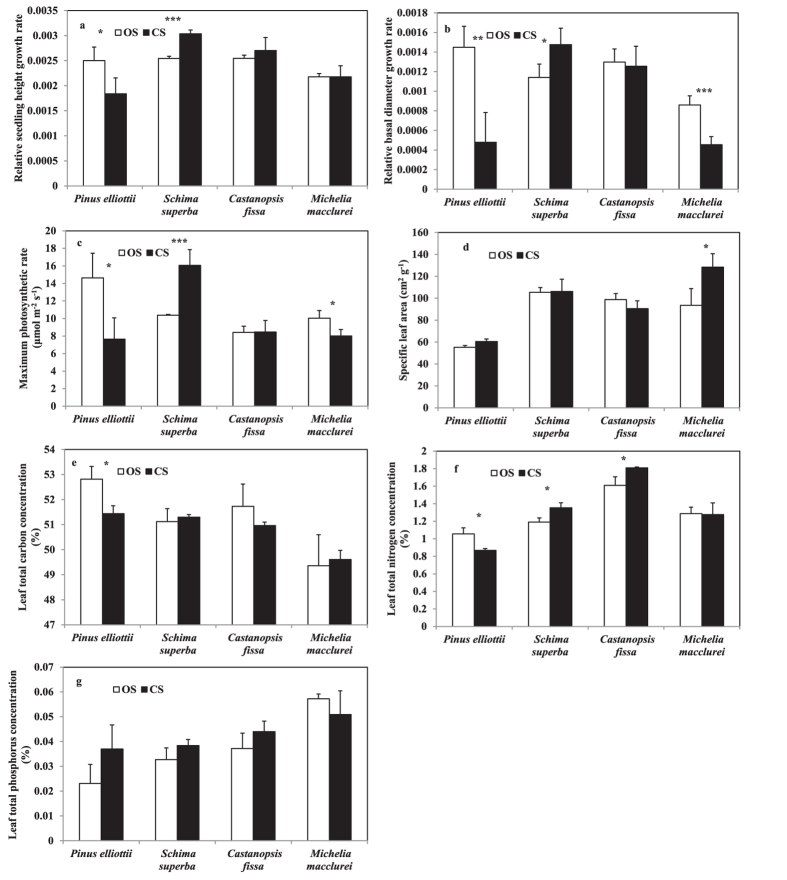
Plant relative seedling height growth rate (**a**), plant relative basal diameter growth rate (**b**), maximum photosynthetic rate (**c**), specific leaf area (**d**), and leaf total carbon (**e**), nitrogen (**f**) and phosphorus (**g**) concentrations. Values are means + SD (n = 5). Single, double and triple asterisks indicate a significant difference between CS and OS plots at p < 0.05, p < 0.005, and p < 0.001, respectively.

Results showed that relative growth rates of seedling height (RGR-H, P < 0.001), and basal diameter (RGR-B, P = 0.001), maximum photosynthetic rates (A_max_, P = 0.023) and specific leaf areas (SLA, P < 0.001) were significantly affected by species, and the interaction between species and treatment (P ≤ 0.023 for all) (Table 1). Meanwhile, RGR-B was also significantly affected by treatment factor (P = 0.012). *Pinus elliottii* showed significantly lowered RGR-H, RGR-B, and A_max_ (P ≤ 0.033 for all) in canopy subplots (CS) than in open subplots (OS) (Fig. 1a–d). Similarly, the RGR-B and A_max_ of *Michelia macclurei* were significantly decreased (P < 0.001, and P = 0.012), but SLA was increased (P = 0.012) when grown in CS than in OS. In contrast, *Schima superba* had much higher RGR-B, RGR-H, and A_max_ (P ≤ 0.015 for all) grown in CS than in OS. These parameters for *Castanopsis fissa*, however, were not significantly different between shrub nurse treatments and controls.

### Leaf main element concentration

Leaf total carbon (C), nitrogen (N), and phosphorus (P) concentrations were significantly affected by species (P < 0.001 for all), but not affected by treatment (P ≥ 0.223 for all) (Table 1). Meanwhile, leaf N concentration was significantly affected by the interactions between species and treatment (P = 0.003). For *P. elliottii*, leaf C and N concentrations were significantly lower (P = 0.008, and P = 0.006) when grown in CS than in OS (Fig. 1e–g). In contrast, for *S. superba* and *C. fissa*, leaf N concentrations were significantly increased when grown in CS than in OS (P = 0.001, and P = 0.009). Leaf P concentrations were not significantly changed between treatment for any of the four species.

### Leaf chemical composition

**Figure 2:**
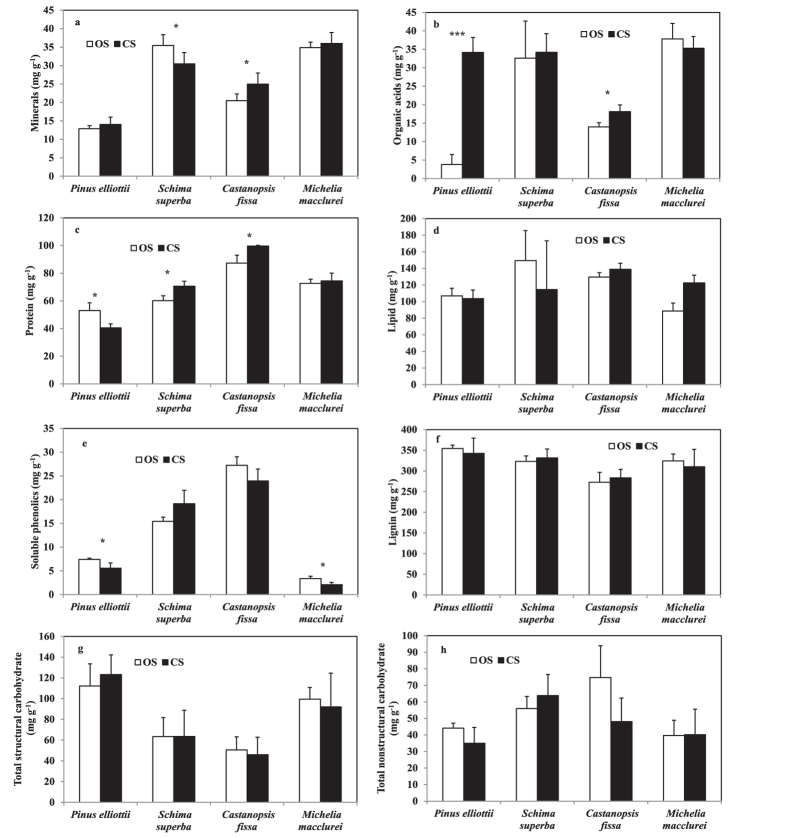
Concentrations of the chemical constituents in leaves of four target plants growing in open subplots (OS) and canopy subplots (CS). (**a**) minerals; (**b**) organic acids; (**c**) proteins; (**d**) lipids; (**e**) soluble phenolics; (**f**) lignin; (**g**) total structural carbohydrates; and (**h**) total non-structural carbohydrates. Values are means + SD (n = 5). Single, double and triple asterisks indicate a significant difference between CS and OS plots at p < 0.05, p < 0.005, and p < 0.001, respectively.

Leaf minerals, organic acids, protein, lipid, soluble phenolics, lignin, TSC and TNC concentrations were significantly affected by species (P ≤ 0.041 for all), but only leaf organic acids and soluble phenolics concentrations were significantly affected by treatment (P = 0.002, and P = 0.023, respectively) (Table 2). Leaf minerals, organic acids, protein, lipid concentrations were also significantly affected by the interactions between species and treatment (P ≤ 0.019 for all). For *P. elliottii* seedlings, leaf organic acids was significantly increased (P < 0.001) but leaf protein (P = 0.018) and soluble phenolics (P = 0.039) were significantly decreased when grown in CS than in OS (Fig. 2). The minerals concentration of *S. superba* was significantly lower (P = 0.046) but protein concentration of this species was significantly higher in CS than in OS (P = 0.025). For *C. fissa*, minerals, organic acids and protein concentrations were all significantly higher when grown in CS than in OS (P ≤ 0.037 for all). For *M. macclurei*, only soluble phenolics were significantly lower when grown in CS than in OS (P = 0.016). Lignin, TSC or TNC concentrations were not significantly affected by shrub nurse effect for any of the tested target species.

### Construction costs and payback time

**Figure 3:**
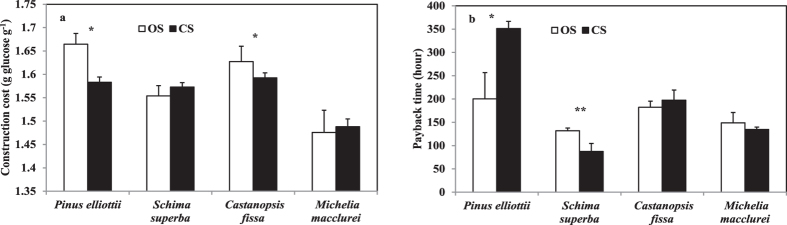
Leaf construction costs (**a**) and payback times (**b**) for four target plants growing in open subplots (OS) and canopy subplots (CS). Values are means + SD (n = 5). Single and double asterisks indicate a significant difference between CS and OS plots at p < 0.05, and p < 0.005, respectively.

CC value and payback time were significantly affected by species (P < 0.001, and P = 0.009), but not significantly affected by treatment and the interaction between species and treatment (Table 1). For *P. elliottii*, CC value was significantly decreased (P = 0.003) but payback time was significantly increased (P = 0.047) when grown in CS than in OS (Fig. 3). Also, CC value of *C. fissa* and payback time of *S. superba* were found to be decreased in CS than in OS (P = 0.044, and P = 0.001). CC values and payback time of *M. macclurei* were not significantly different between treatments.

## Discussion

Overall, our results supported our hypotheses that 1) the growth of the fast growing species (e.g. *P. elliottii* and *M. macclurei*) are suppressed under the canopy of shrub while the more shade tolerant species are facilitated (e.g. *S. superba*); 2) *S. superba* outperforms *P. elliottii* and *M. macclurei* due to its changed foliar chemical composition, unchanged CC values and shortened payback time when facilitated by the shrub.

As revealed by other studies, nurse plant can simultaneously exert both facilitative and competitive effects on target plants seedlings, and the relative dominance of either positive or negative effects largely relies on the traits of tested species^6^,^7^,^10^,^14^. Our previous studies also showed that the differences in target plant growing performances in canopy subplots were mainly caused by their adaptations to light environment^8^,^17^. *S. superba* is a typical late-successional species that adapted to a wide range of light intensity, i.e. it is shade tolerant in juvenile stage but grow in higher light conditions as they mature^13^. In contrast, *P. elliottii*, is a fast growing species, and *M. macclurei* is a light demanding species, thus the canopy shade composed negative growing conditions for their growth during the 3.5-year experiment. Moreover, changes in plant resource use efficiencies may also help explain such differences, i.e. some species outperform others by acquiring limited resources or by using resources more efficiently^12^,^27^. For *M. macclurei*, the lowered photosynthetic rates together with the unchanged leaf C, N, P concentrations under *R. tomentosa* may have decreased photosynthetic energy, nitrogen or phosphorus -use efficiency when grown in canopy subplots. In contrast, *S. superba* can highly increase its resource use efficiencies when nursed by shrub *R. tomentosa*. Thus, we conclude that the shrub nurse plant does not benefit the growth and resource-use efficiency of *P. elliottii* and *M. macclurei* during the 3.5 years. It follows that the former two species may not be suitable for long-term shrubland restoration when the shrub *R. tomentosa* is used as a nurse plant.

Although attempts to understand the mechanisms underlying differences in plant utilization and allocation of assimilated carbon among species have often focused on the role of photosynthesis, process of downstream carboxylation may also provide important information on how carbon assimilation is related to the chemical composition of plant organs^18^. The metabolism of organic acids is fundamentally important at the cellular level for several biochemical pathways, or at the individual level in modulating plant adaptation to the environment^28^. Organic acids are also involved in transporting micronutrients in the transpiration stream in the xylem^29^,^30^. In our study, the highly increased organic acids in foliar tissues of *P. elliottii* and *C. fissa* grown in canopy subplots showed that the nurse shrub coverage may largely improve their cellular metabolism such as participate in the balance of charges formed during the extensive metabolism of anions^28^,^31^.

As a component of functional proteins, structural proteins, and the photosynthetic machinery, nitrogen is an important plant nutrient^32^. In this study, higher leaf nitrogen concentrations were associated with elevated leaf protein contents in *S. superba* and *C. fissa* growing in canopy subplots. Reduced leaf nitrogen contents in *P. elliottii*, in contrast, were associated with decreased protein contents in canopy subplots. There may be a trade-off between investing nitrogen in the photosynthetic apparatus such as chlorophyll rather than in structural compounds such as cell wall proteins^33^,^34^. The proportions of leaf nitrogen partitioned to different nitrogen pools are affected by irradiance^35^, nutrition^36^, and other environmental factors^34^. In this study, as affected by the microenvironment changed by shrub nurse plant, more nitrogen may have been transformed to protein in leaves of *S. superba* and *C. fissa*, while less nitrogen was available for constructing different forms of protein in needles of *P. elliottii* growing in canopy subplots vs. open subplots.

Soluble phenolics are thought to have antioxidant effects in stressed plants^37^. Researchers have proposed that a high concentration of phenolics may protect leaves against high light conditions and may help avoid photosynthetic down-regulation and photoinhibition^38^,^39^. In our study, plants growing in the open rather than canopy subplots may have suffered from excessive radiation. In two of our tested target species, *P. elliottii* and *M. macclurei*, phenolic concentrations were substantially higher in open subplots than in canopy subplots. The increased phenolic concentrations may have protected their photosynthetic apparatus against the excessive energy. These results also indicate that *P. elliottii* and *M. macclurei* can cope with high light conditions and that their establishments in subtropical degraded shrublands may not require shade condition composed by shrub coverage.

The increase in phenolics is mostly associated with lignification because lignin compounds often create barriers between injured and healthy tissues^40^. Unlike previous studies, however, our study did not detect significant differences in lignin concentration in open vs. canopy subplots for any of the four target species. Total structural carbohydrate and structural carbon in plants are mostly involved in morphogenesis of the plant cytoskeleton, and total structural carbohydrate content is relatively stable within a species^41^. If the growth of a plant is limited by photosynthesis, total non-structural carbohydrate may be lower in a declining stand than in a healthy stand^42^. In our study, however, total structural and non-structural carbohydrate concentrations did not differ in open subplots vs. canopy subplots for any of the four species, although there were clear differences in growth and photosynthetic performances in the two subplot types.

Researchers have concluded that there is a compromise in chemical composition between growth and defence within a plant because of limitations in available energy^43^. According to this theory, a plant must choose to invest energy in growth-related processes or in the accumulation of defence-related compounds^44^. For example, species with high growth potential invest more energy in primary compounds (proteins) and less in secondary compounds with a possible defence role (such as phenols or lignin); the opposite is true for species with a low growth potential^18^,^45^. This theory was partially supported by the relationships between chemical compounds and the growth of target species in canopy subplots in the current study. Because the shrub canopy provides a relatively benign micro-environment for other plants, *S. superba* and *C. fissa* seedlings growing in canopy subplots invested more energy in the synthesis of proteins than in the synthesis of defence compounds (e.g. phenolics or lignin). In contrast, *P. elliottii* had decreased levels of proteins and soluble phenolics in canopy subplots, indicating that this target species did not benefit from *R. tomentosa*.

The CC value is an important parameter relative to the carbon budget of plants. It is a “black box”, however, because the underlying mechanisms that produce different values across treatments are unclear^18^. Increasing our understanding of CC values requires additional analyses of the chemical compositions of plants. For example, positive correlations between compounds with high energy costs (e.g. proteins, soluble phenolics, and lignin) and low energy costs (e.g. minerals and organic acids) could buffer variations in CC values and help explain the difference in CC values between treatments 22,46,47. In this study, low levels of high-cost compounds (e.g. proteins) accompanied by high levels of low-cost compounds (e.g. organic acids) contributed to significantly lower CC values for *P. elliottii* growing in canopy subplots than in open subplots. For *S. superba*, because the increased levels of low-cost minerals could not balance the increased levels of high-cost proteins, CC values did not significantly differ between open and canopy subplots. In contrast, the elevated levels of low-cost minerals together with low-cost organic acids resulted in significantly decreased CC values for *C. fissa* seedlings growing in canopy subplots. For *M. macclurei*, except for soluble phenolics, most of the chemical compounds did not significantly differ in canopy vs. open subplots, which resulted in little change in CC values.

Research on leaf CC values and associated traits (e.g. payback time for the carbon investment) has provided insights into carbon acquisition strategies of plants and has therefore helped explain plant growth patterns and population dynamics^48^. Our study also showed that the measurement of a single chemical component or nutrient element might not be able to reveal much about the mechanisms of the plant nurse effects on target species. CC values together with estimated payback time, however, integrate the changes of plant chemical composition and photosynthesis, which may help explain plant response to nurse plants and may enable us to predict long-term growing performances of target plants. The calculated payback time based on CC values for *P. elliottii* was significantly elevated, but that for *S. superba* was decreased when growing in canopy subplots. From the perspective of long-term regional restoration practices (>3.5 years), we believe that *P. elliottii* will require a longer time to construct enough carbon for growth and metabolism when growing in the canopy than when growing in open subplots, while the opposite would be true for *S. superba*. Thus, we suggest that *P. elliottii* should be used solely as a pioneer tree to construct plantations, and that *S. superba* can be more widely used to accelerate the establishment of native plantations using *R. tomentosa* as a nurse plant in subtropical degraded shrublands. We also determined that shrub nurse plant did not shorten the payback time for carbon construction of *C. fissa* or *M. macclurei* seedlings, which means that the two species did not benefit from shrub nurse effect during their establishment on subtropical degraded shrublands.

## Conclusion

Changes in plant chemical composition, construction cost and payback time contribute to the different growing performances of target plants when nursed by shrubs. Among the four tested target species, *S. superba* is the only one that can effectively utilize the ameliorated microclimate constructed by shrubs, thus can potentially form multi-species communities and accelerate the reforestation of degraded subtropical shrublands. The findings of this study showed that the outcomes of shrub nurse effects are highly species- and time-specific because not all species will benefitted from shrub nurse plants at the same time. This study only demonstrated the possible mechanisms on plant nurse effect at a certain time point (3.5 year after the initiation of experiment). Therefore, it is possible that the nurse benefit may there during the early establishment state and gradually dissipate thereafter. Reforestation on degraded ecosystems is a long-term practice. Thus, it is crucial that careful experimental selection of both nursing and target species is assessed using long-term studies before massive practical restoration and plantation efforts.

## Methods

### Study site

This study was initiated in 2007 in a subtropical shrubland located at the Heshan National Field Research Station of Forest Ecosystems (112^o^50’E, 22^o^34’N, Heshan County, Guangdong Province, China). The regional subtropical evergreen broadleaved forest has degraded into a community dominated by shrubs and grasses. The soil type in this region is a typical laterite soil that has been seriously eroded because of a lack of forest coverage. The subtropical monsoon climate in this region is characterized by cool and dry winters and humid summers. The annual precipitation ranges from 1460 to 1820 mm, which mainly occurs as rain between March and August. At the station, the mean annual air temperature is 21.7 °C, and the mean annual solar radiation is 435.75 KJ cm^−2^.

### Plant species

*Rhodomyrtus tomentosa* (Ait.) Hassk. (Myrtaceae) is an evergreen shrub that grows naturally in infertile and acidic soils in tropical and subtropical Asia^49^. *R*. *tomentosa*, which is an indicator of acidic soil and a pioneer on bare land, has a cushion-shaped canopy and can grow as tall as 2 m. Our previous work showed that mature shrubs significantly reduced sunlight radiation and soil temperature, but significantly elevated soil volumetric water content, soil bulk density, as well as soil capillary moisture in subtropical degraded shrublands. *R*. *tomentosa* was therefore selected as a nurse plant for different tree seedlings in this and our previous studies^8^,^9^,^17^.

Four tree species were selected as target species. *Schima superba* Gardn. et Champ (Theaceae), *Castanopsis fissa* (Champ. ex Benth.) Rehd. et. Wils, and *Michelia macclurei* Dandy. are broadleaved evergreen native tree species commonly used for reforestation in tropical and subtropical regions of China. Among them, *S. superba* is a shade tolerant, *C. fissa* is a moderate mesophytic and *M. macclurei* is a light demanding species. A fourth target tree species, *Pinus elliottii* Engelm. is an introduced conifer that can grow as tall as 30 m. It is fast growing and resistant to drought and nutrient-poor soils on degraded hills. This pine species is widely used in reforestation in southern China, because it has ecological values such as water and soil conservation, and economic values such as a source of timber, pulp, nuts and rein^50^. Several researches have documented that pines can be facilitated by shrubs 6,7, however, few previous researches tested the facilitation of native shrub *Rhodomyrtus tomentosa* on the establishment and growth of pine seedlings on subtropical shrubland^51^. In this study, four reforestation species, including three broadleaved species and one conifer species, were selected to test whether they can be well facilitated by shrubs in their initial life history, to identify the underlying mechanisms from carbon allocation aspects, and to instruct regional reforestation practices.

### Experimental design

The detailed information regarding the establishment and design of the experiment were previously reported^12^,^17^ and are also briefly described below. In 2007, we divided the 2-ha subtropical shrubland field site into three blocks. Within each block, four plots (5 m × 5 m) were randomly selected, and each plot was assigned to one of the four target tree species, giving a total of three plots for each target tree species. Within each plot, one 2 m × 2 m subplot was assigned the “canopy” treatment (CS), and one was assigned the “open” treatment (OS). CS subplots were located under the circular edges of the *R. tomentosa* canopies, which were 1.0–1.8 m high in 2007^8^. OS subplots were located in areas without woody plant canopies. Seedlings (6 months old) of *P. elliottii*, *S. superba*, *C. fissa*, and *M. macclurei* were transplanted individually in the CS and OS subplots in 2007; a total of 40 seedlings were planted in each subplot. As reported earlier^17^, two-thirds of the target tree seedlings were harvested for analysis at the end of 2008, leaving 10 to 15 seedlings in each subplot. Although some seedling mortality occurred in 2008, no additional mortality occurred in 2009 or 2010.

### Plant growth, photosynthetic and structural parameters

Relative growth rates of seedling height (RGR-H) and basal diameter (RGR-B) of that target species were based on the height and basal diameter measured in October 2010 and the mean values measured in April 2007, and the RGRs were calculated as final minus initial logarithmic values divided by the time between measurements^16^. Leaf photosynthetic rates (A) of each target species were measured using an LI-6400 portable photosynthesis analyser (LI-COR, Lincoln, NE, USA) on clear days in October 2010; in each subplot, four to five replicate leaves (each from one plant) of each species were used. Maximum photosynthetic rates (A_max_) were obtained at 1500 μmol m^−2^ s^−1^ light intensity with ambient CO_2_ concentration and air humidity. The obtained values for A_max_ (μmol CO_2_ m^−2^ s^−1^) were transformed to A_mass_ (nmol CO_2_ g^−1^ s^−1^) based on specific leaf area (SLA). SLA was calculated as the ratio of leaf area/leaf mass after leaves had been oven dried at 60 °C for 72 h^12^.

### Leaf chemical compositions

After photosynthetic rates were measured, leaves on the same branches of each tree were collected and oven-dried at 60 °C for 72 h. The dried leaves were then ground, passed through a 0.08 mm sieve, and dried again in the oven. Total carbon (C) and nitrogen (N) concentrations in the leaf samples were determined spectrophotometrically with the potassium dichromate oxidation spectrophotometric method and the Kjeldahl method. Phosphorus concentration (P) was determined colorimetrically after HClO_4_-H_2_SO_4_ digestion^52^. Nitrate-N contents of leaf samples were measured colorimetrically with salicylic acid^53^. Ash content was determined by combusting 1 g of plant material in a muffle furnace at 550 °C for 6 h and then weighing the residue. Ash alkalinity was determined acidimetrically by titration^54^.

Another set of oven-dried leaf samples (1g) was extracted with a solution of water, methanol, and chloroform in a volumetric ratio of 1:2:2^55^. The extracts in the chloroform phase were dried with a rotary evaporator, and the residue (total lipids) was weighed. Soluble carbohydrates were measured in the methanol-water phase using anthrone reagent^56^. The soluble phenol contents were also determined colorimetrically in the methanol-water phase with Folin-Ciocalteus reagent^57^. After extraction with the water, methanol, and chloroform mixture, the residues of the leaf samples were boiled in 3% HCl (v/v) for 3 h. Insoluble sugars were subsequently analysed in the supernatants^56^. The final residues after boiling with HCl were used for another round of determination of carbon and nitrogen concentration using the potassium dichromate oxidation spectrophotometric method and the Kjeldahl method, respectively^52^.

### Chemical calculations

Total mineral, protein, and organic acid concentrations in target plant leaf samples were estimated using the following equations^58^:


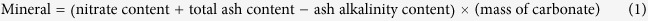



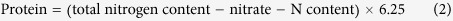



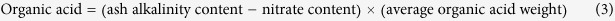


The final residue after extraction was considered to be a mixture of lignin and TSC. Lignin concentration was calculated with the carbon and nitrogen concentration and assuming that the carbon concentration in the (hemi)cellulose complex was 444 mg g^−1^ and that the carbon concentration in lignin was 640 mg g^−1^ 21. CC values of target plant leaf samples were calculated with the following formula^59^:


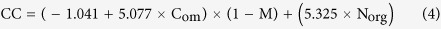


where CC is the construction cost (g glucose g^−1^), C_om_ is the organic carbon concentration (g g^−1^), M is the total mineral content (g g^−1^), and N_org_ is the organic nitrogen concentration (g g^−1^). Because the quantity of ammonium and nitrate taken up by the different tree species was unknown, these CC values should be considered as maximum values.

Payback time was estimated as CC/A_mass_ by transforming the unit of CC from g glucose g^−1^ to nmol C g^−1^ and by transforming the unit of A_mass_ from nmol CO_2_ g^−1^ s^−1^ to nmol C g^−1^ h^−1^. Payback time was calculated per hour rather than per day, because the diurnal radiation period changes during the growing season. As a consequence, the estimated payback time is considered to be the theoretically minimum amortization period^52^,^60^.

### Statistical analyses

We used IBM SPSS Statistics 19.0 for statistical analyses. Results are presented as means + standard deviation (SD). For each target species, differences between OS and CS subplots in plant growth and photosynthetic parameters, CC value, chemical composition, and payback time were analysed with one-way ANOVAs. Two-way ANOVA was applied to determine the effect of species (4 levels) and treatments (2 levels) on plant growth and photosynthetic parameters, CC value, chemical composition, and payback time.

## Additional Information

**How to cite this article**: Liu, N. *et al.* *Schima superba* outperforms other tree species by changing foliar chemical composition and shortening construction payback time when facilitated by shrubs. *Sci. Rep.* **6**, 19855; doi: 10.1038/srep19855 (2016).

## Figures and Tables

**Table 1:** P values from two-way ANOVA statistics for the effects of species (4 levels) and treatment (2 levels) on plant growth parameters, maximum photosynthetic rate, specific leaf area, and leaf total carbon, nitrogen and phosphorus concentrations.

Source of Variation	RGR-Seedling height	RGR-Basal diameter	Maximum photosynthetic rate	Specific leaf area	Leaf carbon concentration	Leaf nitrogen concentration	Leaf phosphorus concentration
Species	0.000	0.001	0.023	0.000	0.000	0.000	0.000
Treatment	0.713	0.012	0.211	0.291	0.223	0.332	0.797
Species × Treatment	0.012	0.002	0.005	0.023	0.241	0.003	0.179

**Table 2:** P values from two-way ANOVA statistics for the effects of species (4 levels) and treatment (2 levels) on CC value, payback time, and chemical compositions.

Source of Variation	CC value	Payback time	Minerals	Organic acids	Protein	Lipid	Phenolics	Lignin	TSC	TNC
Species	0.000	0.009	0.000	0.000	0.000	0.000	0.000	0.007	0.001	0.041
Treatment	0.266	0.357	0.828	0.002	0.242	0.896	0.023	0.867	0.760	0.187
Species × Treatment	0.087	0.240	0.019	0.000	0.001	0.000	0.076	0.553	0.750	0.319
